# Structure-based network analysis predicts pathogenic variants in human proteins associated with inherited retinal disease

**DOI:** 10.1038/s41525-024-00416-w

**Published:** 2024-05-27

**Authors:** Blake M. Hauser, Yuyang Luo, Anusha Nathan, Ahmad Al-Moujahed, Demetrios G. Vavvas, Jason Comander, Eric A. Pierce, Emily M. Place, Kinga M. Bujakowska, Gaurav D. Gaiha, Elizabeth J. Rossin

**Affiliations:** 1 Harvard Medical School, Boston, MA USA; 2 Harvard Medical School, Department of Ophthalmology, Massachusetts Eye and Ear, Boston, MA USA; 3 Ragon Institute of Mass General, MIT, and Harvard, Cambridge, MA USA; 4 Division of Gastroenterology, Massachusetts General Hospital, Boston, MA USA

**Keywords:** Disease genetics, Molecular medicine, Genetics research

## Abstract

Advances in gene sequencing technologies have accelerated the identification of genetic variants, but better tools are needed to understand which are causal of disease. This would be particularly useful in fields where gene therapy is a potential therapeutic modality for a disease-causing variant such as inherited retinal disease (IRD). Here, we apply structure-based network analysis (SBNA), which has been successfully utilized to identify variant-constrained amino acid residues in viral proteins, to identify residues that may cause IRD if subject to missense mutation. SBNA is based entirely on structural first principles and is not fit to specific outcome data, which makes it distinct from other contemporary missense prediction tools. In 4 well-studied human disease-associated proteins (BRCA1, HRAS, PTEN, and ERK2) with high-quality structural data, we find that SBNA scores correlate strongly with deep mutagenesis data. When applied to 47 IRD genes with available high-quality crystal structure data, SBNA scores reliably identified disease-causing variants according to phenotype definitions from the ClinVar database. Finally, we applied this approach to 63 patients at Massachusetts Eye and Ear (MEE) with IRD but for whom no genetic cause had been identified. Untrained models built using SBNA scores and BLOSUM62 scores for IRD-associated genes successfully predicted the pathogenicity of novel variants (AUC = 0.851), allowing us to identify likely causative disease variants in 40 IRD patients. Model performance was further augmented by incorporating orthogonal data from EVE scores (AUC = 0.927), which are based on evolutionary multiple sequence alignments. In conclusion, SBNA can used to successfully identify variants as causal of disease in human proteins and may help predict variants causative of IRD in an unbiased fashion.

## Introduction

As genetic sequencing has become increasingly available and less costly, a growing number of patients with clinical presentations of suspected genetic origin are undergoing targeted or whole-exome sequencing. Despite improved accessibility, the genetic basis of disease for a considerable proportion of these patients remains unclear following sequencing^1^. Inherited retinal diseases (IRD), whereby rod and cone photoreceptors degenerate, are a group of Mendelian disorders that represent an important cause of vision loss^2^. With the advancement in the availability of genetic testing^3–6^ and the lower cost of exome sequencing, IRD is a field with increasing promise and possibility for gene therapy interventions. However, among patients with an IRD, ~30% do not have a clear genetic basis despite classic retinal changes and a decrease in visual and retinal function^7^, making them ineligible candidates for treatment. For these patients, additional tools are needed to better define genetic variants that are not among the group of known causal variants (i.e., variants of uncertain significance—VUS).

Numerous computational tools that aim to predict the phenotype of genetic variants have been described^8–10^, many of which were trained on existing variant classifications or combine multiple metrics that use this type of training data^11–19^. These data were limited by sparsely available annotations^20^, and previous studies suggest that some of these algorithms may also have considerable false discovery rates^21,22^. Furthermore, many of these algorithms are limited by circularity, with duplication of variants in the training and test datasets as well as variants from the same protein within the training and test datasets^23^. The clinical applicability of these approaches has been limited as a result^23,24^. Another class of methods uses sequence conservation to estimate the likelihood that a particular variant will have a deleterious phenotypic effect^25,26^. Sequence conservation is an important feature to consider (and provides independent information from protein structure) but can be an imperfect proxy for the functional importance of a particular amino acid position within a protein of interest; this has been previously demonstrated within the context of both model and human proteins^27,28^ and also the human immunodeficiency virus (HIV), which has a per-base mutation rate ~10^4^ times that of the human genome and thus serves as a model for an accelerated rate of genomic evolution^29–31^. Some of these approaches also leverage recent advances in machine learning, which results in limited post hoc transparency regarding the basis for a particular estimated probability of pathogenicity and is also subject to circularity and over-fitting^26,32^. Functional in vitro assays, such as a high throughput assay for the *RHO* gene, can help to characterize individual genetic variants^33^. However, these crucial experiments are not always feasible on a large scale for the entire set of patient-genotype combinations, given the need for appropriate experimental setup, tissue-type, and readout for the particular variant of interest.

To add to these approaches, we developed structure-based network analysis (SBNA) which leverages the application of network theory to protein structure data with the goal of quantifying local residue connectivity, bridging interactions, and ligand proximity in order to identify amino acid residues that are topologically important^29,34^. Using x-ray crystallography and cryogenic electron microscopy (cryo-EM) data, it models proteins as networks of connected amino acids to quantitatively estimate the topological importance of each amino acid as it relates to others in the protein, protein complex, or protein–ligand interaction. This approach is distinct from prior computational tools that use structural information because it is not reliant on pre-defined secondary structure elements; rather, it analyzes the crystallized tertiary structure of the folded protein as a network of weighted inter-residue interactions. Additionally, this approach does not require training on pre-labeled phenotypic data which means that it can provide a metric that is specifically focused on first-principle structural information. SBNA has been previously used to identify highly mutationally constrained amino acid residues and CD8^+^ T cell epitopes in HIV and severe acute respiratory syndrome coronavirus 2 (SARS-CoV-2)^29,35^. In the case of HIV, these epitopes were found to be preferentially targeted by individuals able to suppress HIV replication in the absence of antiretroviral therapy^29^. Within the context of SARS-CoV-2, highly networked regions have resisted ongoing sequence evolution during the pandemic and thereby may be capable of conferring broad T cell-mediated protection against sarbecovirus infection^35^.

In light of these successes with highly mutable viruses, we chose to apply SBNA to human proteins which exist within a complex system of biological interactions. Such an application would potentially aid in better understanding which genetic variants discovered through sequencing are causal of disease. We first verified this approach in well-studied human proteins: breast cancer gene 1 (BRCA1), GTPase HRas (HRAS), phosphatase and tensin homolog (PTEN), and mitogen-activated protein kinase 1 (ERK2). We then sought to investigate whether this approach could generate meaningful results for IRD proteins of interest to estimate the phenotypic impact of missense variants, given that such a sizable number of patients with IRDs harbor VUSs. Therefore, we assessed the performance of SBNA in IRD protein-coding genes and their respective missense variants described in ClinVar, a large public database of reported pathogenic and benign genetic variation. Finally, we addressed a cohort of 63 affected subjects for whom a genetic cause of their condition has yet to be discovered to investigate the use of SBNA in real-world clinical scenarios.

## Results

### Structure-based network analysis accurately identifies missense variants in human proteins associated with in vitro loss of function and pathogenic clinical phenotypes

**Fig. 1:**
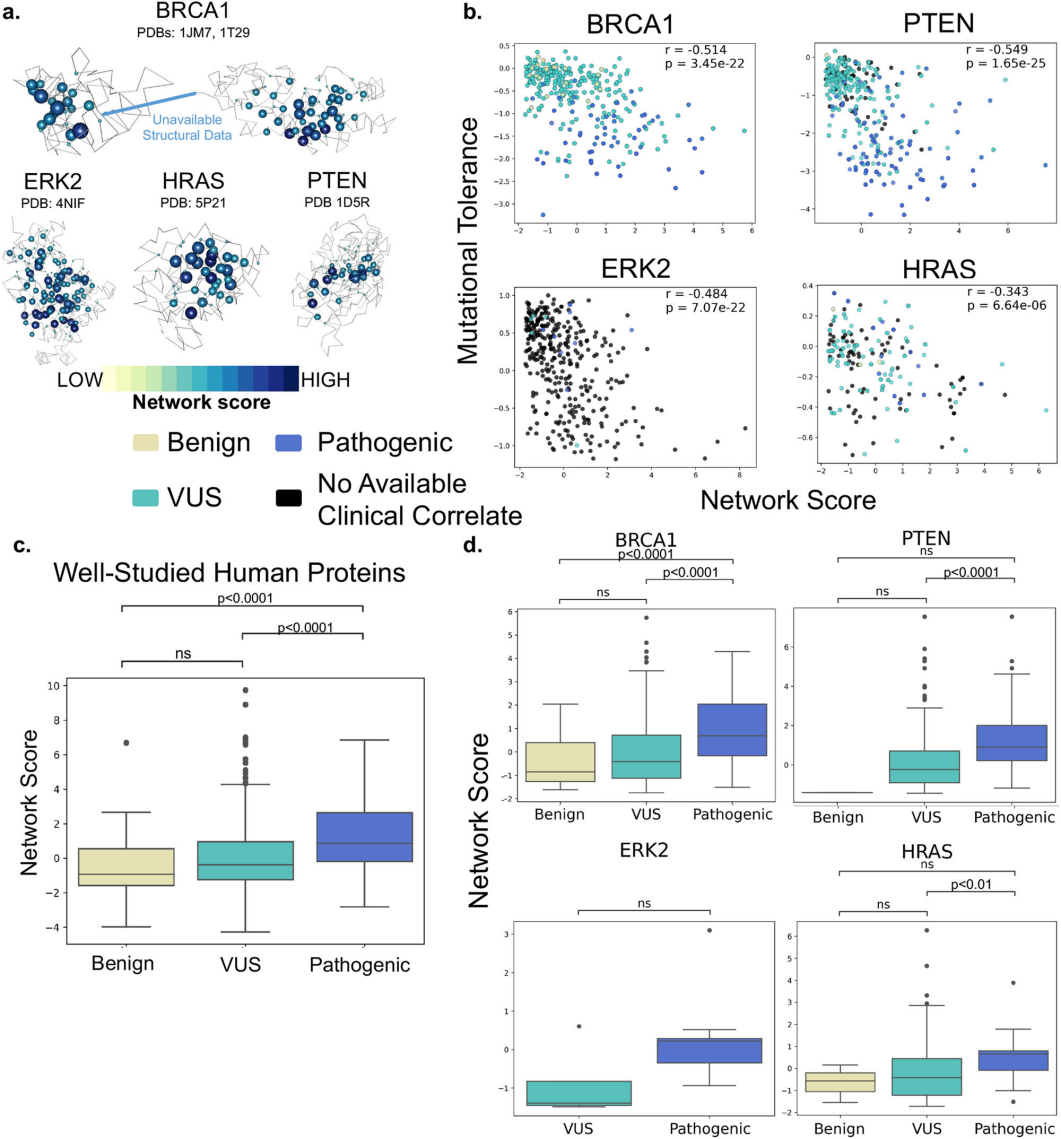
Residue network score correlates with mutation intolerance and distinguishes pathogenic variants from benign variants in well-studied human proteins. **a** Structural representations show network scores at each residue. Sphere radius and color corresponds to network score magnitude at a particular position. **b** Comparison between functional data from saturation mutagenesis experiments and network scores, with Spearman correlation coefficients and p-values displayed for each plot. Points are colored based on available clinical phenotype data. **c** Pooled comparison between network scores for variants with available clinical phenotype data for all four well-studied human proteins. **d** Individual comparisons between network scores for variants with available clinical phenotype data for all four well-studied human proteins.

To evaluate whether SBNA could be meaningfully applied to human proteins, we first applied the approach to four well-studied human proteins—BRCA1, HRAS, PTEN, and ERK2—which were selected for analysis due to the availability of high-quality structural data. In addition, these proteins had published in vitro saturation mutagenesis experiments, which allowed us to extract the functional consequence of all missense variants and quantify mutational tolerance^36–39^. We next generated network scores for all amino acid residues in the available protein databank (PDB) files (Fig. 1a) and evaluated the correlation with mutational tolerance (Fig. 1b). All four proteins showed a strong inverse correlation between mutational tolerance and network score, which was consistent with previous findings for viral proteins and other model proteins^29^. Of note, only 17% of BRCA1 has been crystallized (the N- and C-terminal ends), but SBNA scores still performed reasonably well (Spearman correlation coefficient −0.514, *p* = 3.45e-22) despite limited structural data.

We next compared network scores to pathogenicity categorizations derived from human data using the ClinVar and gnomAD genetic databases for these same four proteins (Fig. 1c). Missense variants in all subsequent analyses were categorized with respect to human clinical data in line with the American College of Medical Genetics and Genomics/Association of Molecular Pathology (ACMG/AMP)^24^ as benign (encompassing “benign” or “likely benign” within ClinVar), VUS or pathogenic (encompassing “pathogenic” or “likely pathogenic” within ClinVar). We restricted our analysis to ClinVar missense variants with at least two-star level evidence, and gnomAD was used to identify additional relatively benign missense variants (variants with at least 250 alternative allele counts across 100,000 individuals). Across all four proteins, network scores assigned to pathogenic variants were significantly greater than those assigned to benign variants (median network scores for benign missense variants −0.936 and pathogenic variants 0.866, *p* = 1.836e-5, Fig. 1c). Scores assigned to VUSs fell in between those assigned to benign and pathogenic variants. These trends were observed consistently within individual proteins, though smaller sample sizes limit statistical power (Fig. 1d). Overall, network scores correlate with available clinical phenotype data for the four well-studied human proteins (Spearman correlation coefficient 0.228, *p* = 2.116e-23), suggesting that SBNA can be meaningfully applied to human proteins. Of note, we found that SBNA seems to capture structural properties beyond simple solvent accessibility (i.e., proximity to the core) because relative solvent accessibility (RSA) scores show less correlation with mutational tolerance scores than did SBNA (Supplementary Fig. 1).

### SBNA predicts variants in IRD genes associated with pathogenic clinical phenotypes

**Fig. 2:**
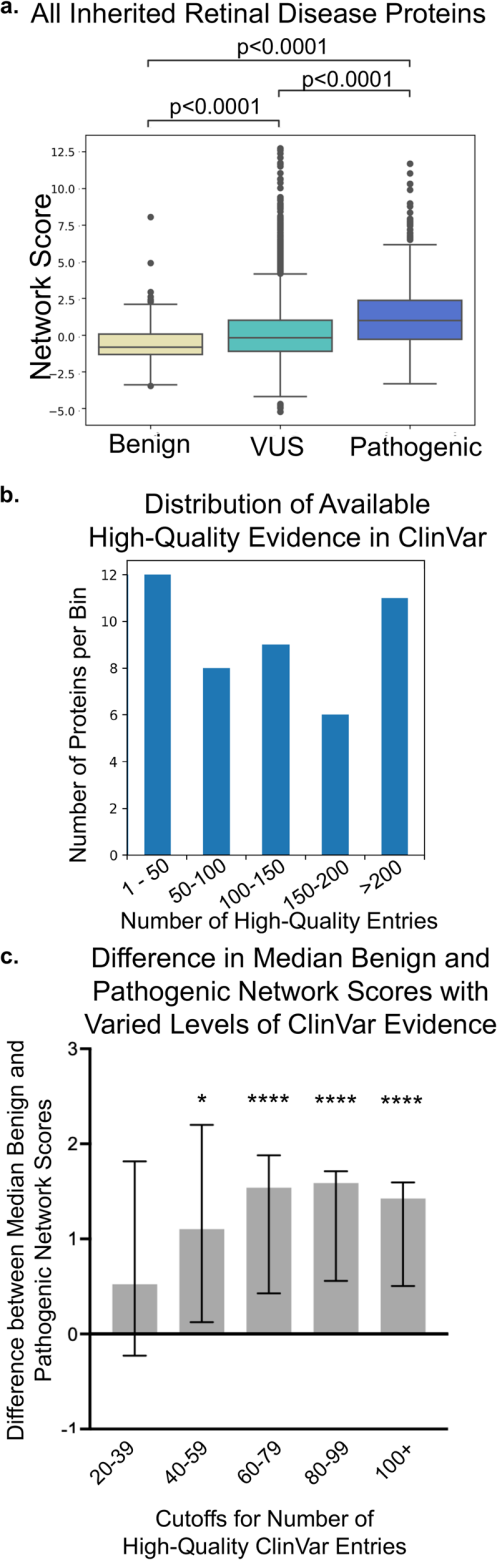
Structure-based network analysis identifies pathogenic variants in inherited retinal disease proteins. **a** Pooled comparison between network scores for variants with available clinical phenotype data for all 47 inherited retinal disease proteins. **b** Distribution of available high-quality evidence in ClinVar across all 47 inherited retinal disease proteins. The bins reflect increasing numbers of high-quality entries in ClinVar, and the height of each bar reflects the number of proteins in each category. **c** Comparison between median benign and pathogenic network scores assigned to variants with available high-quality evidence in ClinVar, grouped by level of available high-quality evidence for each inherited retinal disease protein. Stars above columns indicate statistical significance (*p < 0.05, **p < 0.01, ***p < 0.001, ***p < 0.0001). The statistical significance of the difference in network scores between benign and pathogenic variants is lost between 20 and 40 high-quality ClinVar evidence entries.

Having validated SBNA on four canonical, well-studied human proteins, we then applied SBNA to additional human proteins. We analyzed the relationship between network scores and pathogenicity designations from high-quality ClinVar variants and benign gnomAD variants for the 47 human genes associated with IRDs, for which high-quality structural data is available for the encoded protein. This dataset includes both membrane proteins (e.g., ABCA4 and RHO) as well as cytoplasmic proteins (e.g., RPE65 and RPGR) (Supplementary Table 1). We found that pathogenic variants were assigned significantly greater network scores compared to both benign variants and VUSs (median benign −0.841, median VUS −0.188, median pathogenic 0.947 *p* = 3.140e-29 for benign vs. pathogenic, *p* = 1.753e-60 for VUS vs. pathogenic; Fig. 2a and Supplementary Fig. 2), which is similar to the pattern observed for the canonical human proteins. Because the number of high-quality ClinVar entries for missense variants in each of the 47 IRD-associated proteins varies considerably (Fig. 2b), we wanted to evaluate whether the difference between median benign and pathogenic network scores remained statistically significant even for proteins with limited variant data available in ClinVar. We grouped proteins by the amount of available high-quality entries in ClinVar and calculated the difference between the median benign and pathogenic network scores across each group of proteins. The magnitude of these differences was robust in the setting of differing levels of available clinical data across genes and was detectable down to 40 high-quality entries per gene (Fig. 2c).

### Incorporating network scores and BLOSUM62 scores successfully predicts variant pathogenicity

**Fig. 3:**
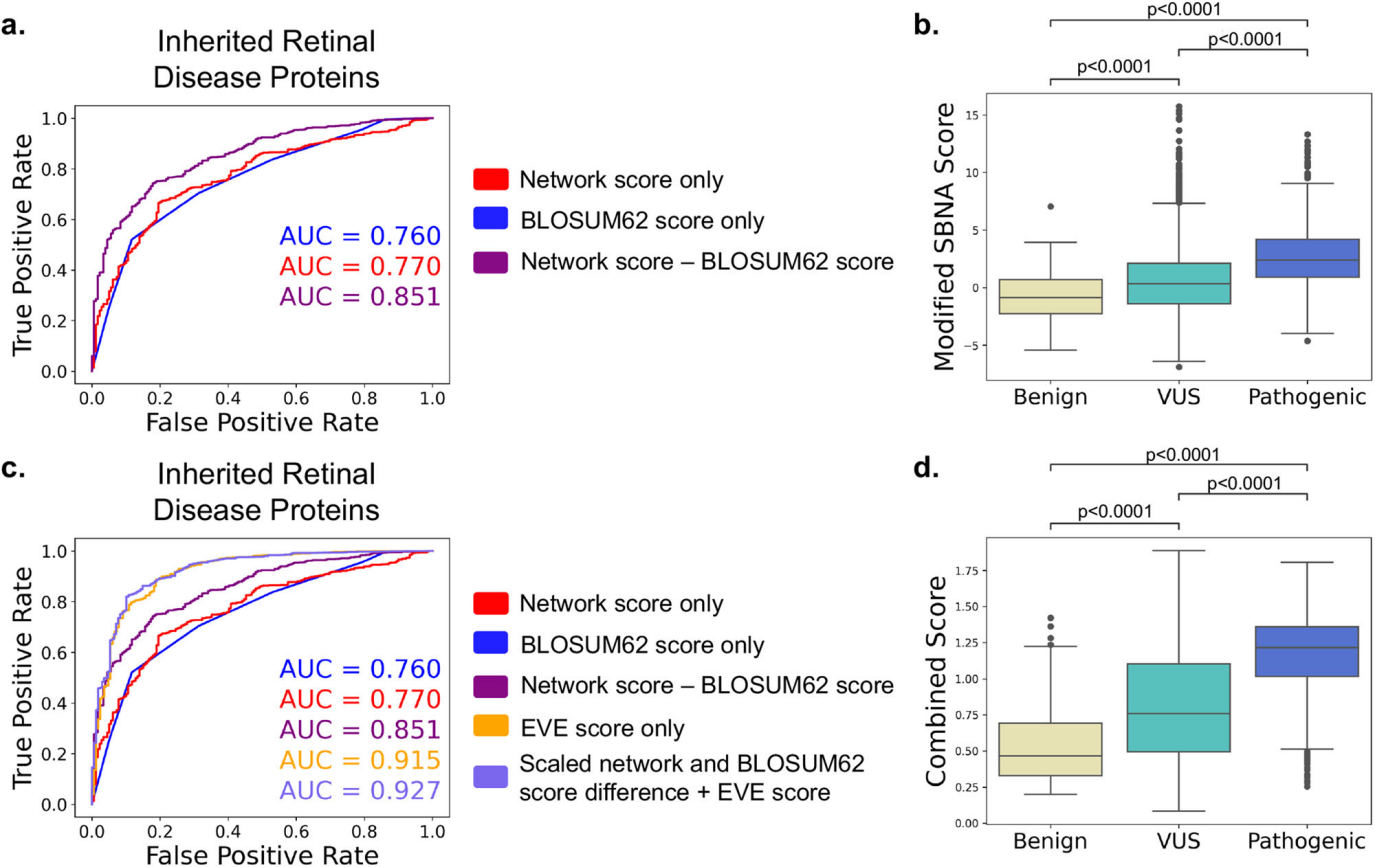
Modeling using SBNA and BLOSUM62 is superior to SBNA network scores and BLOSUM62 scores alone. **a** ROC curves for network scores alone (red), BLOSUM62 scores alone (blue), and the difference between network scores and BLOSUM62 scores (purple). ROC curves were determined using all variants with available clinical phenotype data for all 47 inherited retinal disease proteins. AUC values are shown for each curve. **b** Pooled comparison between the difference between network scores and BLOSUM62 scores for variants with available clinical phenotype data for all 47 inherited retinal disease proteins. **c** ROC as shown in (**a**) with added comparison to EVE scores (orange) and the sum of EVE scores and the scaled difference between network scores and BLOSUM62 scores (light purple). ROC curves were determined using all variants with available clinical phenotype data for all 47 inherited retinal disease proteins. AUC values are shown for each curve. **d** Pooled comparison between the sum of EVE scores and the scaled difference between network scores and BLOSUM62 scores (“combined score”) for variants with available clinical phenotype data for all 47 inherited retinal disease proteins.

To move from aggregate statistics to prediction of pathogenicity using network scores, we constructed a modified score that incorporates not only the SBNA network score but also the degree of chemical and side chain dissimilarity between the reference and mutant amino acid at that position (since missense variants vary in this regard). To capture the latter effect, we subtracted the BLOSUM62 matrix score from the SBNA score (which we will now refer to as the modified SBNA score) to allow for a distinction between non-conservative substitutions (e.g., ILE → TRP) and conservative ones (e.g., ILE → LEU)^40^. BLOSUM62 scores for non-synonymous substitutions range from −4 to 3, while 95% of the raw SBNA scores range from −2.88 to 4.97; thus, the two metrics are on similar scales and can be combined with simple subtraction. We then calculated receiver operating characteristic (ROC) curve statistics for high-quality ClinVar variants and benign gnomAD variants based on the modified SBNA score. Modified SBNA scores predicted variant pathogenicity (AUC 0.851) and outperformed network scores alone, BLOSUM62 scores alone, and RSA scores alone (Fig. 3a, b and Supplementary Fig. 3). We tested multivariable logistic regression modeling with 500 iterations of a 70/30% train-test split as well as a leave-one-out approach using the labels derived from high-quality ClinVar and gnomAD variants and found similar performance to the raw scores (AUC 0.835, Supplementary Figs. 3, 4). Given the advantage that using the raw scores has over a trained approach (which can be subject to poor phenotypic labeling and data circularity^20,23^), all downstream clinical applications described here use the raw modified SBNA score.

### Comparison of SBNA to existing methods reveals similar performance without dependence on phenotype labels or evolutionary sequence data

We set out to compare SBNA to three tools that are built from different underlying data: Polyphen2, AlphaMissense, and EVE. Polyphen2 is a widely used computational prediction tool for variant pathogenicity^41^. It is important to note however that PolyPhen2 is used by the ACMG/AMP to assess pathogenicity designations in databases such as ClinVar and HumDiv, so using ClinVar as ground truth might overestimate the performance of PolyPhen2. AlphaMissense^32^ incorporates structural data from AlphaFold2^42^, protein language modeling, and evolutionary multiple sequence alignments into a machine-learning model using ClinVar labels to train. Similar to PolyPhen2, there is a risk of over-fitting and circularity with AlphaMissense. EVE is a variant pathogenicity approach which relies only on evolutionary sequence data rather than clinical labeling for model training and is thus not subject to circularity, similar to SBNA^26^. EVE performs well on two of the well-studied human proteins (Spearman correlation ranging from −0.478 to −0.513, benign versus pathogenic *P* < 0.05 for all; Supplementary Fig. 5); EVE predictions are not available for ERK2. To minimize bias, all algorithms were tested on an independent dataset of 2800 rare variants derived from patients with an IRD who were seen at MEE, though of note, ground truth is still determined using the ClinVar database in accordance with the clinical standard in the field. Thus, methods that train on ClinVar must be interpreted with caution. The modified SBNA scores were compared to results generated using PolyPhen2 trained on two different datasets, HumDiv and HumVar^12,41^, as well as EVE scores^26^. All methods showed a significant difference between benign and pathogenic variants, and the modified SBNA scores correlated with the scores from other methods (Supplementary Figs. 6A, B, 7A, B, Spearman correlation coefficient range 0.510–0.571, *p* < 5e-24 for all). With a threshold of 1.5 for modified SBNA scores, the sensitivity was 0.548, specificity 0.908, positive predictive value 0.963, and negative predictive value 0.312.

ROC curves were generated by testing each of the methods on the dataset of 2800 variants present in MEE patients (AUC range: 0.788 [modified SBNA], 0.829–0.833 [PolyPhen2], 0.819 [AlphaMissense], and 0.809 [EVE]), Supplementary Figs. 6C, 7C). The modified SBNA, PolyPhen2, AlphaMissense, and EVE performed similarly, though PolyPhen2 and AlphaMissense scores may be inflated due to training on ClinVar pathogenicity labels.

### Combining SBNA and EVE outperforms all methods individually

The correlation and prediction results suggest that structural information and sequence conservation provide distinctly important insight into pathogenicity, and thus, incorporating orthogonal metrics into a single score may help to improve model correlation with phenotypic benchmarks. We thus sought to use the two unbiased methods (modified SBNA and EVE). The modified network scores were scaled and added to EVE scores to create a combined score with a range of 0 to 2 (as EVE scores fall between 0 and 1, and SBNA scores were scaled based on the maximum and minimum values across all proteins such that they fell between 0 and 1 before adding to the EVE scores). When applied to the 2800 rare variants from MEE patients as well as the 47 IRD genes, this combined score distinguished pathogenicity (Supplementary Fig. 7D, benign vs. pathogenic *p* = 3.056e-17; Fig. 3d, benign vs. pathogenic *p* = 8.839e-69) and outperformed all other models with an AUC of 0.859 (Supplementary Fig. 7E) for the 2800 variants and 0.927 (Fig. 3c) for the IRD genes. With a threshold of 1.0 for the combination score, the sensitivity was 0.765, specificity 0.899, positive predictive value of 0.976 and negative predictive value of 0.416. While we note that EVE alone performed quite well (AUC = 0.915), adding the modified SBNA improves performance and, importantly, unlike EVE alone, offers a direct structural explanation for the mechanism through which variation may affect phenotype.

### Model incorporating the modified SBNA scores identifies putative disease-causing variants with an unclear genetic basis for clinical disease

A significant percentage of patients with clinical presentations consistent with IRD lack an identified genetic basis for their phenotype, and this is also observed for patients who receive care from the Inherited Retinal Disease Service at MEE. We therefore evaluated genetic data from 3621 probands with a clinical diagnosis of an IRD based on visual acuity, visual field testing, clinical exam, fundus autofluorescence imaging, optical coherence tomography and electroretinogram in individuals who underwent targeted or whole-exome sequencing. Mitochondrial causes of IRD were excluded. Missense variants of interest were defined using variant ranking software^43^ and residence in one of the studied 47 IRD genes. There were 2948 unique variants identified and categorized as either “pathogenic”, “likely pathogenic”, “VUS”, or “benign” based on a known variant consequence in the literature using ACMG/AMP criteria^44^ and ClinVar designations^20^ (Supplementary Fig. 8). Variants were further categorized in the context of individual patients as “likely causal” if they were pathogenic or likely pathogenic, the zygosity was consistent with known modes of inheritance, and the clinical presentation was consistent with the known consequence of the affected gene. Of all the reviewed patients, 455 patients carried variants of interest in the 47 IRD genes. Before applying SBNA, 357 were found to have variants that were “likely causal”, while 63 patients harbored one or more VUSs that prohibited a molecular diagnosis. The remaining 35 patients had non-missense variants or variants within a region that lacked available structural data and were therefore excluded.

**Fig. 4:**
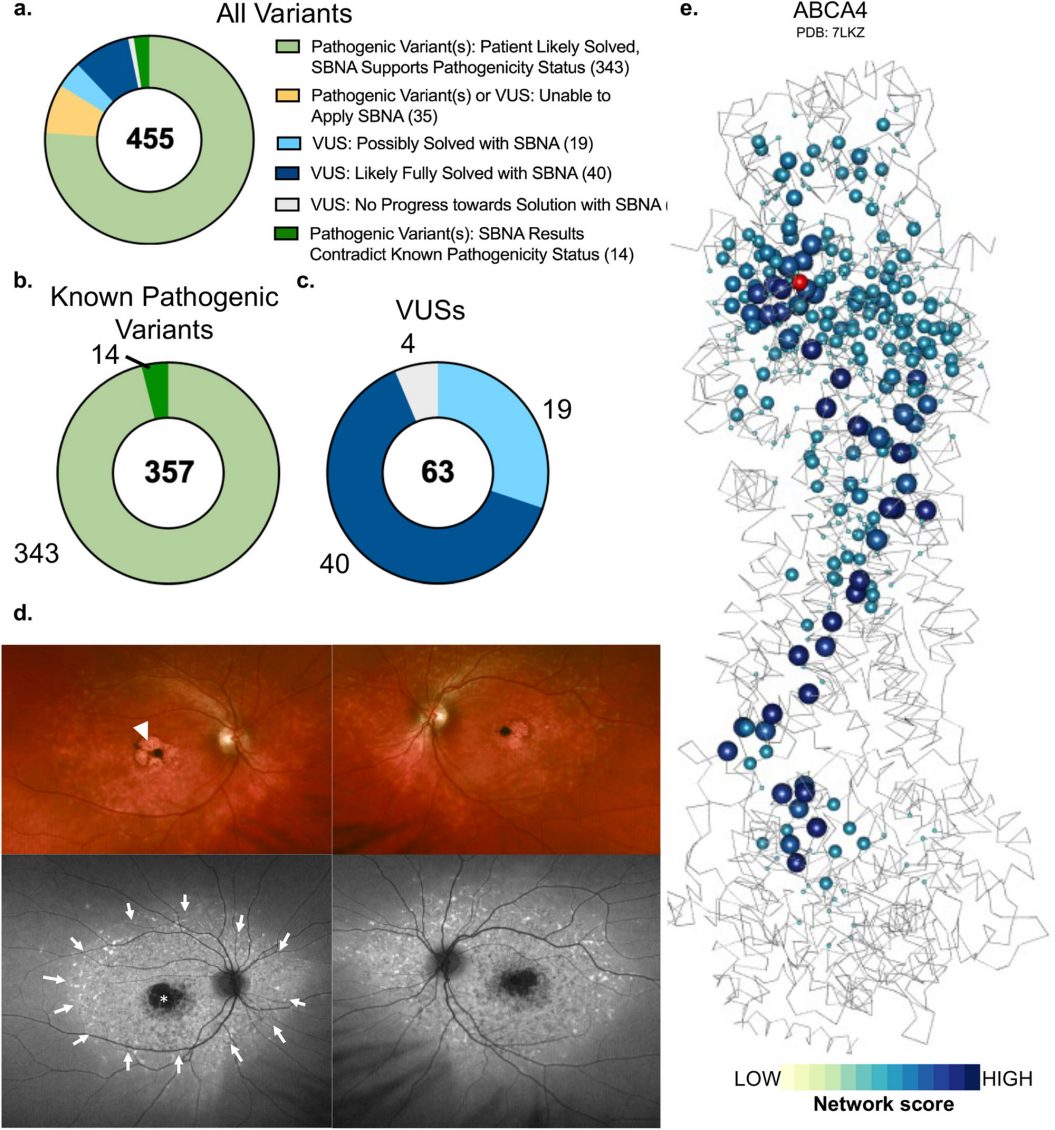
SBNA helps identify pathogenic variants in patients with inherited retinal disease. **a** Categorization of results from the application of modified structure-based network analysis (SBNA) to a dataset of possibly solving patient variants. Results were further subdivided into those from patients with known putative genetic causes of disease (**b**) and those from patients with only VUSs in known inherited retinal disease-associated genes (**c**). **d** Representation of network scores for a sample structure with putative solving genetic variants. Sphere radius corresponds to network score magnitude at a particular position. A patient with clinical evidence of ABCA4 disease (**d**) as evidenced by bilateral foveal pigmentary changes (arrowhead) on color fundus photo and bilateral RPE atrophy (star) and hyper autofluorescent flecks throughout the posterior pole on fundus autofluorescence imaging (arrows) but with no complete genetic explanation was fully solved using SBNA which highlighted two variants (Pro1380Leu and Arg1097Ser) that score highly in the ABCA4 protein structure (**e**). Arg1097Ser was a VUS and is indicated in red within the structure.

We generated pathogenicity predictions for the 2,948 IRD gene variants discovered in the probands (Fig. 4a). Variants receiving a modified SBNA score of >1.5 (calibrated using probability estimates from the regression modeling) were deemed pathogenic. Scores were considered in the context of the identified genetic variants in known IRD genes for each patient with a clinical presentation consistent with IRD. Variants were matched with any phenotypic data available in ClinVar to roughly benchmark the pathogenicity scores. Similar to the ClinVar analysis of IRD genes, there were observable differences between the modified SBNA scores assigned to known pathogenic variants as compared to benign variants and VUS/variants without any available clinical data (benign median −1.478, VUS median −0.656, pathogenic median 2.148; benign vs. pathogenic *p* = 1.713e-30).

For the 357 patients who harbored known pathogenic variants sufficient to cause disease, the modified SBNA scores were concordant with these pathogenicity categorizations in 96.0% of cases (Fig. 4b). For the 63 patients with VUSs as categorized by ACMG/AMP standards^44^ and/or ClinVar, the modified SBNA scores offered support for a genetic cause of disease for 40 patients (23 unique variants, Fig. 4c). By contrast, EVE scores offered support for a genetic cause of disease in 25 patients (20 in common with SBNA), PolyPhen2 scores trained on HumVar offered support in 33 patients (27 in common with SBNA), and AlphaMissense offered support in 40 patients (34 in common with SBNA). Combined EVE and modified SBNA scores offered support in 23 patients (20 in common with SBNA), but this analysis was limited because EVE does not provide scores for 15 patients. In the 15 patients where modified SBNA scores suggested a putative causative variant but EVE scores did not, one patient had a variant for which no EVE score was provided in the database, and the remainder had at least one candidate variant with an EVE score below the predicted pathogenicity threshold. Modes of inheritance included autosomal recessive (in combination with a known pathogenic variant or a second VUS with a high estimated probability of pathogenicity; *n* = 15), autosomal dominant (*n* = 2), and X-linked recessive (*n* = 5). For example, a patient with autosomal recessive *ABCA4*-related disease was found to have variants p.(Pro1380Leu) (known pathogenic) and p.(Arg1097Ser) (VUS). The p.(Arg1097Ser) variant scored highly (SBNA score 3.672, BLOSUM62 score -1, score difference 4.672), suggesting it is likely pathogenic and thus completing the genetic solution for this patient. Similarly, the VUS p.(Cys302Tyr) in *RPGR* was found in a hemizygous patient with phenotypic findings consistent with X-linked IRD and also scored highly (SBNA score 2.154, BLOSUM62 score -2, score difference 4.154) (Supplementary Fig. 9). For 19 patients, the modified SBNA scores contributed towards identifying a possible but not completely solved genetic cause, such as only one heterozygous VUS receiving a strong score. Finally, for the four remaining unsolved patients, SBNA was not possible due to lack of crystal structure data for those portions of the IRD-associated proteins or due to the presence of non-missense variants.

### AlphaFold2 can improve structural coverage for SBNA

**Fig. 5:**
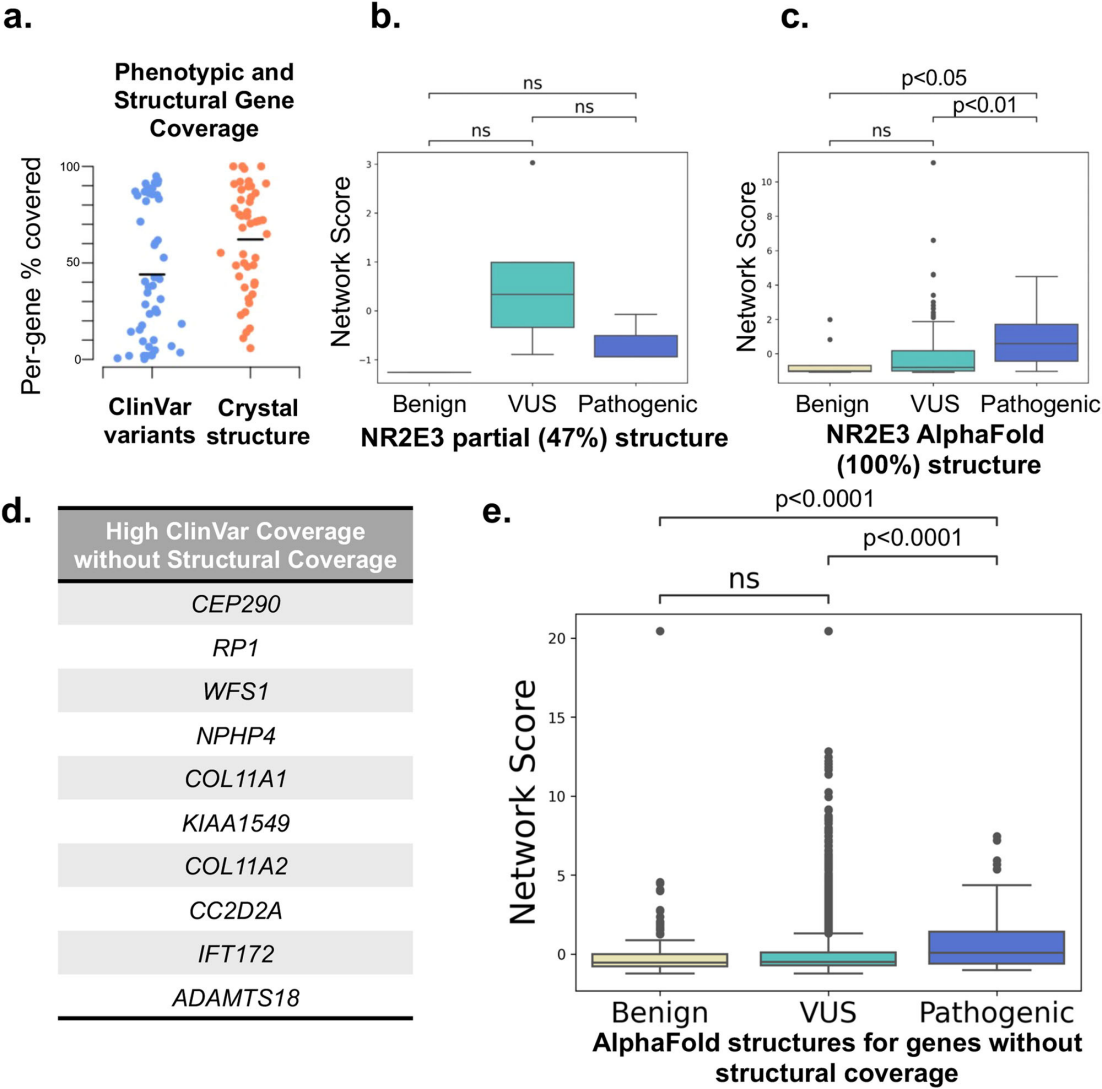
AlphaFold2 improves coverage for SBNA. **a** The percentages of high confidence ( ≥ 2 stars) ClinVar variants for each of 47 IRD genes captured with SBNA are widely distributed (blue); the per-protein percentages of solved structure are widely distributed (orange). **b** NR2E3 only has structural data available for a single domain as part of a chimera. SBNA scores for NR2E3 correlate poorly with pathogenicity because the structure is partial and non-physiologic, but (**c**) the performance improves when using AlphaFold2 to model the full human protein. **d** Ten IRD-associated genes without available structural data were selected, and **e** SBNA scores were calculated for the full AlphaFold2 structures.

Despite evidence of strong performance when applied to IRD-associated proteins, SBNA remains broadly limited by the availability of high-quality structural data for proteins of interest. This structural coverage must also overlap with the availability of high-quality phenotypic data from ClinVar, limiting the scope of analysis (Fig. 5a). Applying AlphaFold2 may provide a path toward overcoming this limitation. For example, NR2E3 was excluded from the set of 47 IRD genes because the only available structural data is from a chimera formed between one domain of NR2E3 and an unrelated bacterial protein (PDB: 4LOG). SBNA performs poorly on this non-physiologic structure with relatively poor coverage of NR2E3 (47%) (Fig. 5b). However, when SBNA is applied to the full AlphaFold2-generated human NR2E3 structure, performance improves considerably (Fig. 5c).

To further establish that AlphaFold2 can help to overcome the limited availability of high-quality structural data for SBNA, we selected ten IRD-associated genes without available structural data that had a considerable amount of data in ClinVar (Fig. 5d). We performed SBNA on the AlphaFold2-generated structures for these genes and found a significant difference between the SBNA scores assigned to benign and pathogenic variants (*p* = 1.201e-7; benign median −0.539, VUS median −0.491, pathogenic median 0.076) (Fig. 5e). However, the magnitude of difference between the median benign and pathogenic network scores was decreased compared to SBNA performed on the structural data from the 47 IRD-associated genes. Still, these results suggest that AlphaFold2 could potentially be useful in expanding the applicability of SBNA, although it is not clear that the quality of this analysis would be superior to that performed on experimentally generated structural data.

## Discussion

In this study, we applied SBNA to human proteins and demonstrated that the resulting residue network scores, especially when augmented with BLOSUM62 substitution scores, correlate strongly with missense variants that cause functional deficit or clinical disease. We leveraged those scores, in combination with BLOSUM62 substitution scores, to generate estimates of the likelihood that a particular missense variant was pathogenic. Using these estimates, we were able to nominate putative genetic solutions for 40 patients with clinical evidence of IRD. These results suggest that SBNA provides meaningful insights for patients with an unclear genetic basis for their clinical symptoms, particularly for patients with inherited retinal diseases. Importantly, we note that this method is based purely on structural principles rather than training on labeled outcome data, which means that it not only provides an unbiased prediction of pathogenicity but also the mechanism of pathogenicity (i.e., structural change) is proposed.

Numerous computational tools for the prediction of variant pathogenicity have been developed^15,32,45–56^. Virtually all of these tools use sequence conservation and protein secondary structure or domain information. These two categories of features can work well, especially when used in conjunction with one another. However, SBNA is distinct in that it captures the structural topology of individual amino acid residues in the context of the protein architecture and does not rely on pre-existing annotations. Importantly, unlike all existing tools, the performance of SBNA does not seem to rely on a training step involving thousands of phenotype-genotype combinations or multiple sequence alignment. As shown, the raw SBNA score combined with BLOSUM62 (without training) achieves an AUC of 0.851 on the dataset of 47 IRD proteins. Furthermore, the biophysical basis for SBNA scores can be analyzed on an atomic level. This transparency facilitates downstream applications, such as the consideration of possible gene therapy targets, and is not available within models that heavily leverage machine-learning approaches^26,32^. The predictive value of SBNA can be further augmented by incorporating EVE scores, which are ultimately based on evolutionary multiple sequence alignments^26^, to achieve an AUC of 0.927. This suggests that incorporating multiple orthogonal metrics may strengthen predictive models.

Applying SBNA may provide additional insight into patient candidacy for both approved and developmental gene therapy treatments. By identifying candidate pathogenic variants with high confidence, SBNA could be used to nominate a limited subset of variants for expedited in vivo validation to fast-track delivery of appropriate therapies to patients. Broadly, the patients that may benefit clinically from SBNA fall into three categories. The first are patients who may be a candidate for gene-specific, variant-agnostic therapies, such as the FDA-approved *RPE65*-targeted gene therapy voretigene neparvovec^57^. This also applies to any gene with variant-agnostic therapies in ongoing clinical trials. A second category of patients could potentially benefit from variant-specific gene editing therapies, such as those facilitated by CRISPR-Cas9-mediated non-homologous end joining, which are currently under development for *CEP290*^58^. The third category of patients with IRD who may benefit from SBNA are not yet candidates for any existing clinical therapies. However, using SBNA on a large scale to identify candidate disease-causing variants may be informative for nominating new genes for variant-agnostic therapy development and new variants for gene editing therapy development in order to maximize potential patient benefit.

This approach is limited by the availability of high-quality structural data, a requirement for SBNA^29^, though we note that any tool that proposes to use structural data will be reliant on this. Numerous proteins, including the majority that correspond to known genetic variants associated with IRD, lack available x-ray crystallography or cryo-EM structures altogether. In some cases, structures are available but are not of sufficient resolution to facilitate downstream network analysis. Furthermore, this approach will only be applicable to variants that result in a negative structural change. Other types of variants – such as splice site variants – will not be captured with this approach. However, given that this tool only requires protein structure data, we expect that the performance of SBNA will continue to improve. Software that leverages artificial intelligence to predict protein structure, such as AlphaFold2 or RoseTTAFold, have already emerged to help compensate for the lack of available structural data^42,59^. We have demonstrated that SBNA can be meaningfully applied to some of these in silico-generated structures, which may provide a path towards overcoming the limitation of high-quality structural data availability going forward.

In conclusion, SBNA is a new tool to estimate the likely extent of the phenotypic impact of missense variants by assessing the topological important of affected residues to protein structure. We demonstrated that this technique could be meaningfully applied to human proteins and showcased the use of SBNA in IRD patients who lack a clear genetic diagnosis. These types of insights could contribute to the design of novel gene therapies targeted at implicated genetic variants^57,60,61^.

## Methods

### Structural data

All structural data were downloaded from the Protein Data Bank^34^. Individual accession numbers for the well-studied human proteins and inherited retinal disease proteins are listed in Supplementary Table 1. For the non-human benchmark proteins, the same files were used as previously described^29^. In cases where multiple human structures were available, the highest oligomeric state of the protein with a resolution of ~3 angstroms or better was used. In cases where no human structure was available, the structure of one or more homologs was used. If multiple chains were present within the PDB file due to crystal packing rather than true oligomerization, only one of these chains was used for SBNA. Solvent and water molecules were removed from all PDB files prior to SBNA, but ligands and protein binding partners were included in the analysis. Only the protein of interest was designated to have a network score calculated by SBNA.

### Structure-based Network Analysis

Structure-based network analysis was used to calculate network scores as previously described^29,35^. The details of this method have been described previously. As before, in cases where multiple conformations of a structure were used, network scores were averaged for each amino acid position. In cases where no human structure was available, the structures of one or more non-human homologs as listed in Supplementary Table 1 were used as templates in Modeler to generate a predicated human structure. Relative solvent accessibility (RSA) was calculated using DSSP files^62^ and previously published maximum solvent accessible surface area values^63^. Code to perform structure-based network analysis is publicly available via Zenodo (10.5281/zenodo.2597484).

### Data analysis and visualization

Data analysis was performed using Python (version 3.8.2), with visualizations generated using the “matplotlib” package. Logistic regressions were performed using the “glm” package in R (version 4.0.4). Intercepts were set to zero for all logistic regression models. SBNA and logistic regression results for all tested variants are available via the Harvard Dataverse (10.7910/DVN/YEEPDY). Network score visualizations were generated in R (version 4.0.4) using the “rgl” package to implement OpenGL. The backbone centroid (centroid of nitrogen, alpha carbon, and carbon) positions were plotted along x, y, and z axes, and nodes were colored and given sphere radii corresponding to network scores. The protein structure backbone was then plotted, connecting the alpha carbons. Plotted structures were manually rotated, and two-dimensional views of interest were downloaded for inclusion.

### Phenotype data

Clinical phenotype data was downloaded from ClinVar. Clinical evidence with a two-gold star level designation (meaning that “two or more submitters with assertion criteria and evidence [or a public contact] provided the same interpretation”) or better was included in the published analyses^20^. Pathogenicity designations from ClinVar were binned into Benign (“Benign”, “Benign/Likely benign”, “Likely benign”), VUS (“Uncertain significance”, “not provided”, “Conflicting interpretations of pathogenicity”), and Pathogenic (“Pathogenic”, “Pathogenic/Likely pathogenic”, “Likely pathogenic”) categories for analysis.

Additionally, allelic variation data from gnomAD^64^ was considered for each gene. Loci with at least 250 available allelic variants in gnomAD were considered benign variants. Results from ClinVar and gnomAD for each gene were considered in combination for these analyses.

### Functional data

Functional data for the four well-studied human proteins—BRCA1^36^, ERK2^39^, PTEN^37^, and HRAS^38^—that had been previously published was used for this analysis. In cases where functional scores were assigned to multiple amino acid variants at the same position (e.g., if different functional scores were calculated for Ala101Pro and Ala101Gln), the arithmetic mean of all functional scores at that position was used.

### Patient data

Patient data was gathered from among those presenting to the Inherited Retinal Disorders Service at Massachusetts Eye and Ear between the 1980s and 2020s. Appropriate written informed consent was obtained from all included patients, and approval was granted by the Mass General Brigham/Massachusetts Eye and Ear Institutional Review Board Protocol Number 2019P001098. This study was approved by the local institutional review board and adhered to the Declaration of Helsinki. Informed consent was obtained from all individuals on whom clinical data and genetic testing were performed.

### Genetic analyses

Pre-existing genetic solutions were available for all of the 3621 IRD cases, where genetic diagnoses for 3018 cases was obtained by targeted next-generation sequencing approaches^43^ or whole genome sequencing, and the remaining solutions were available from prior single-strand conformation polymorphism (SSCP) analysis and Sanger sequencing. Sequence data was aligned to the hg38 genome build, and the subsequent variant calling, annotation, and analyses were performed as described^43^.

### Model Comparisons

SBNA was compared to results from PolyPhen2^41^ (trained on both HumDiv and HumVar and EVE^26^, with scores from both models generated as described in their respective publications. EVE scores were downloaded from https://evemodel.org; PolyPhen2 scores were generated using the “batch query” function at http://genetics.bwh.harvard.edu/pph2/. To analyze the likelihood of pathogenicity, SBNA scores, EVE scores, and PolyPhen2 scores (trained on HumVar) were calculated for a set of 2800 variants. Cutoffs for each model were as follows: modified SBNA score >1.5 (corresponding to an approximate pathogenicity probability of 75% in the logistic regression model), EVE score ≥0.65, and PolyPhen2 designation of “probably damaging”. The “combined score” was generated by scaling modified SBNA scores to fall between 0 and 1 based on the minimum and maximum values across all proteins and adding them to EVE scores. A threshold of 1.0 was selected as this corresponds to the sum of the minimum pathogenic EVE score (0.65) and the scaled version of the minimum pathogenic modified SBNA score (1.5, which scales to 0.37) with two significant figures.

### Statistical analysis

Statistical analysis, including the generation of ROC curves, was performed using the “scipy.stats” package in Python as well as GraphPad Prism (version 9). Comparisons between three or more categories were made using the non-parametric Kruskal-Wallis test with Dunn’s post hoc analysis corrected for multiple comparisons with a Bonferroni correction. Comparisons between the two categories were performed using the non-parametric Mann–Whitney *U*-test. The correlation between the two datasets was calculated using non-parametric Spearman correlation coefficients. Spearman correlation coefficients between network scores and ClinVar pathogenicity designations was calculated by assigning 0 to benign variants, 1 to VUS, and 2 to pathogenic variants.

### Reporting summary

Further information on research design is available in the Nature Research Reporting Summary linked to this article.

### Supplementary information

Supplementary Materials

Reporting summary

## Data Availability

ClinVar, gnomAD, and Protein Data Bank data were publicly available and can be accessed using the gene names and unique identifiers in Supplementary Table 1. Patient data from Massachusetts Eye and Ear cannot be shared publicly as specified by the Institutional Review Board due to concerns regarding possible identifiability of patients with relatively rare genetic conditions. Qualified researchers may be able to access the data via collaboration and data usage agreement by contacting the corresponding author.

## Code availability

Code to perform structure-based network analysis is publicly available via Zenodo (10.5281/zenodo.2597484).
