# Supplementary material for: Structure-based network analysis predicts pathogenic variants in human proteins associated with inherited retinal disease
Source: NPJ Genom Med. 2024 May 27;9:31. doi: 10.1038/s41525-024-00416-w (PMC11130145; doi:10.1038/s41525-024-00416-w)
Supplement: Supplementary file 1 — Supplementary Materials [file 41525_2024_416_MOESM1_ESM.pdf]

Supplementary Materials for:

Title: Structure-based network analysis predicts pathogenic variants in human proteins associated with inherited retinal disease

Authors: Blake M. Hauser, PhD[1]; Yuyang Luo, PhD[2]; Anusha Nathan, BS[3]; Ahmad Al-Moujahed, MD, PhD, MPH[2]; Demetrios Vavvas, MD, PhD[2]; Jason Comander MD, PhD[2]; Eric A. Pierce, MD, PhD[2]; Emily M. Place, MS[2]; Kinga M. Bujakowska, PhD[2]; Gaurav D. Gaiha, MD, DPhil[3,4], Elizabeth J. Rossin, MD, PhD[2]

[1] Harvard Medical School, Boston, MA

[2] Department of Ophthalmology, Massachusetts Eye and Ear, Harvard Medical School, Boston, MA

[3] Ragon Institute of Mass General, MIT, and Harvard, Cambridge, MA

[4] Division of Gastroenterology, Massachusetts General Hospital, Boston, MA

Corresponding author:

Elizabeth J. Rossin, MD, PhD

Department of Ophthalmology

Massachusetts Eye and Ear

243 Charles St, Boston, MA 02114, USA

E-mail address: elizabeth_rossin@meei.harvard.edu

a.

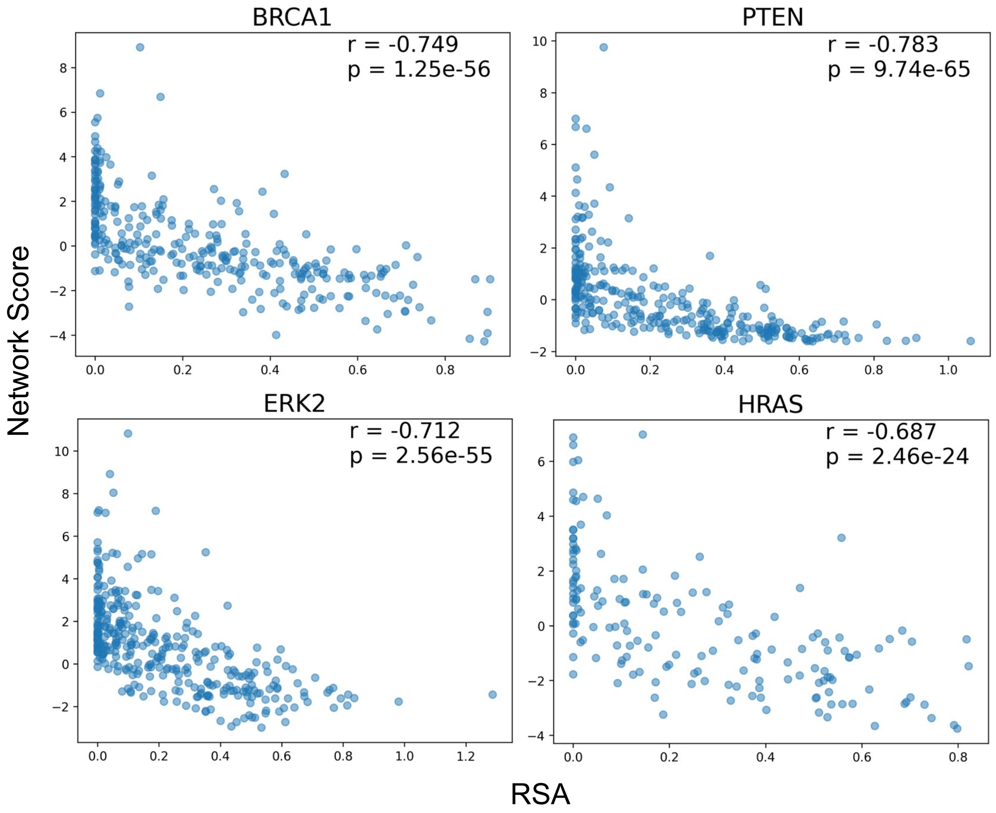
BRCA1
r = -0.749
p = 1.25e-56
PTEN
r = -0.783
p = 9.74e-65
ERK2
r = -0.712
p = 2.56e-55
HRAS
r = -0.687
p = 2.46e-24
Network Score
RSA

b.

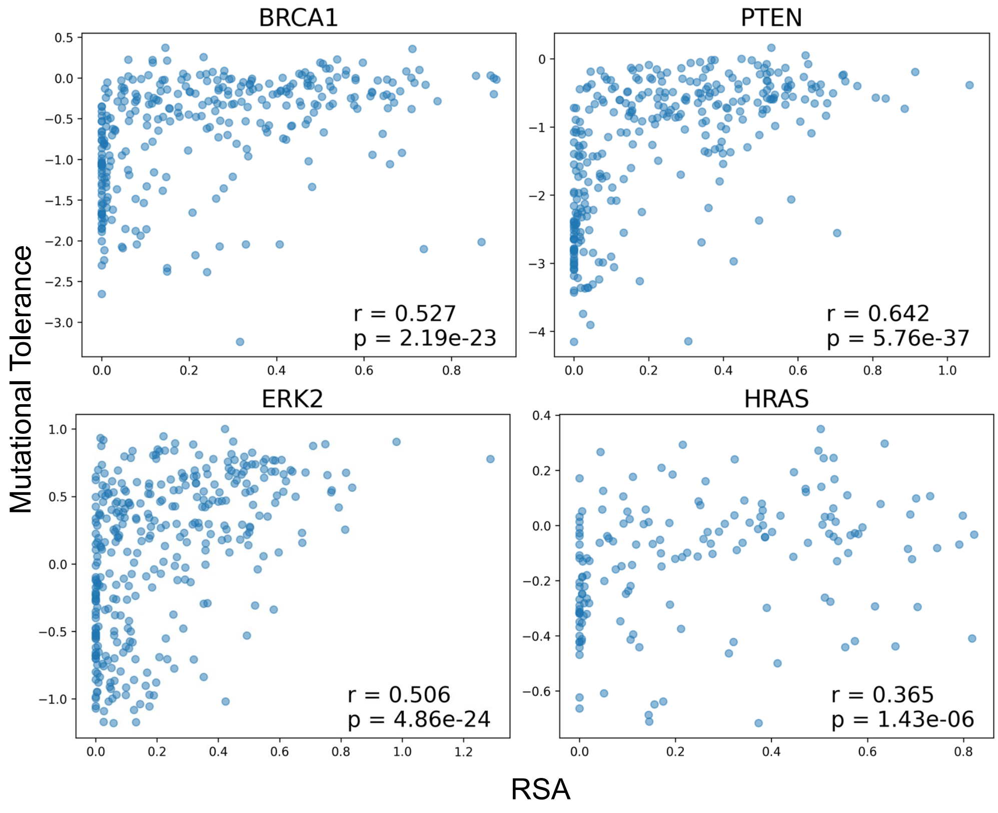
BRCA1
r = 0.527
p = 2.19e-23
PTEN
r = 0.642
p = 5.76e-37
ERK2
r = 0.506
p = 4.86e-24
HRAS
r = 0.365
p = 1.43e-06
Mutational Tolerance
RSA

Supplementary Figure 1. Network scores and mutational tolerance correlate with relative solvent accessibility. (A) Network scores and **(B)** *in vitro* saturation mutagenesis functional scores (corresponding to mutational tolerance) correlate with RSA. Spearman correlation coefficients and p-values are displayed for each plot.

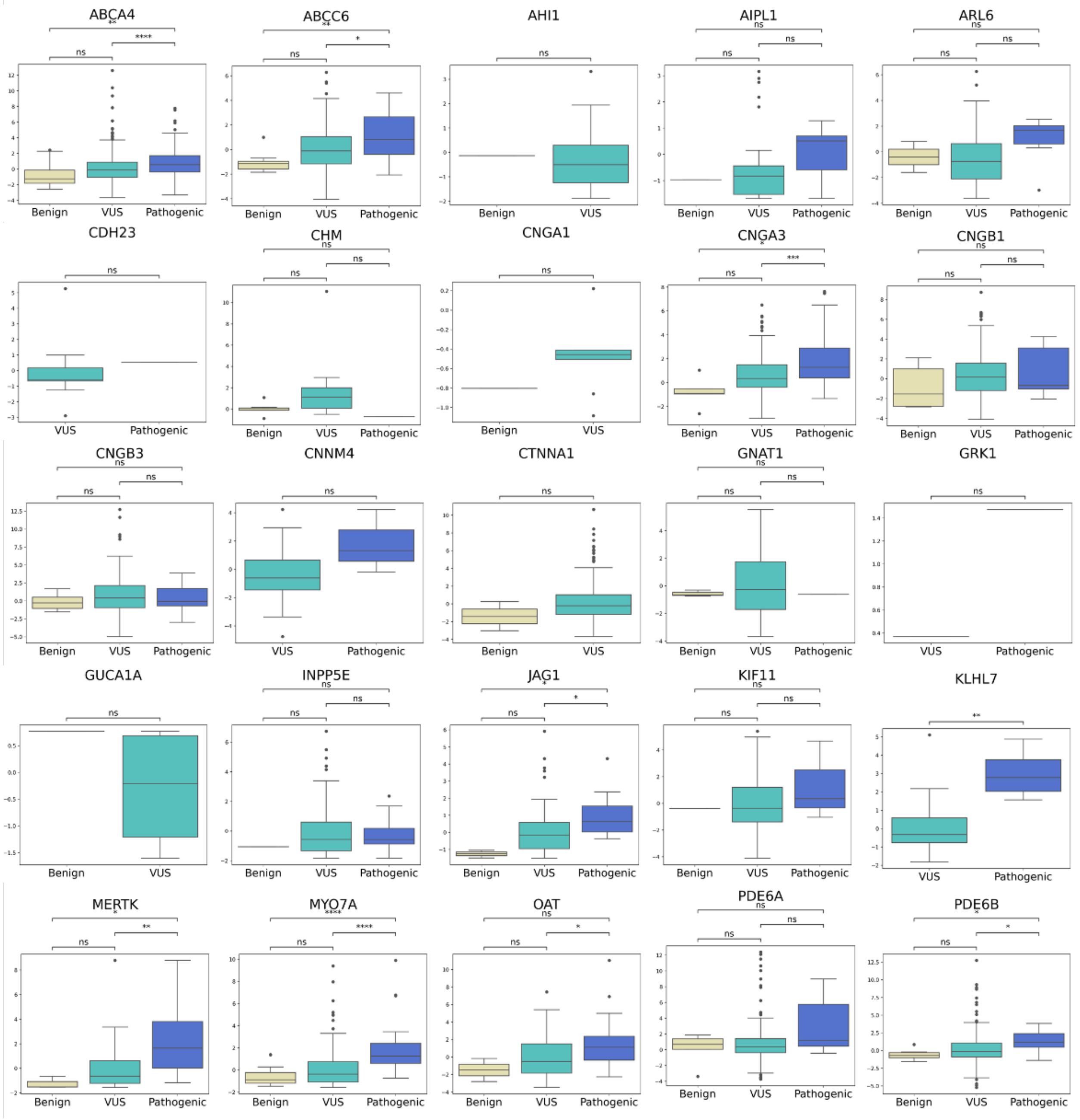
ABCA4
Benign VUS Pathogenic
ABCC6
Benign VUS Pathogenic
AHI1
Benign VUS
AIPL1
Benign VUS Pathogenic
ARL6
Benign VUS Pathogenic
CDH23
VUS Pathogenic
CHM
Benign VUS Pathogenic
CNGA1
Benign VUS
CNGA3
Benign VUS Pathogenic
CNGB1
Benign VUS Pathogenic
CNGB3
Benign VUS Pathogenic
CNNM4
VUS Pathogenic
CTNNA1
Benign VUS
GNAT1
Benign VUS Pathogenic
GRK1
VUS Pathogenic
GUCA1A
Benign VUS
INPP5E
Benign VUS Pathogenic
JAG1
Benign VUS Pathogenic
KIF11
Benign VUS Pathogenic
KLHL7
VUS Pathogenic
MERTK
Benign VUS Pathogenic
MYO7A
Benign VUS Pathogenic
OAT
Benign VUS Pathogenic
PDE6A
Benign VUS Pathogenic
PDE6B
Benign VUS Pathogenic

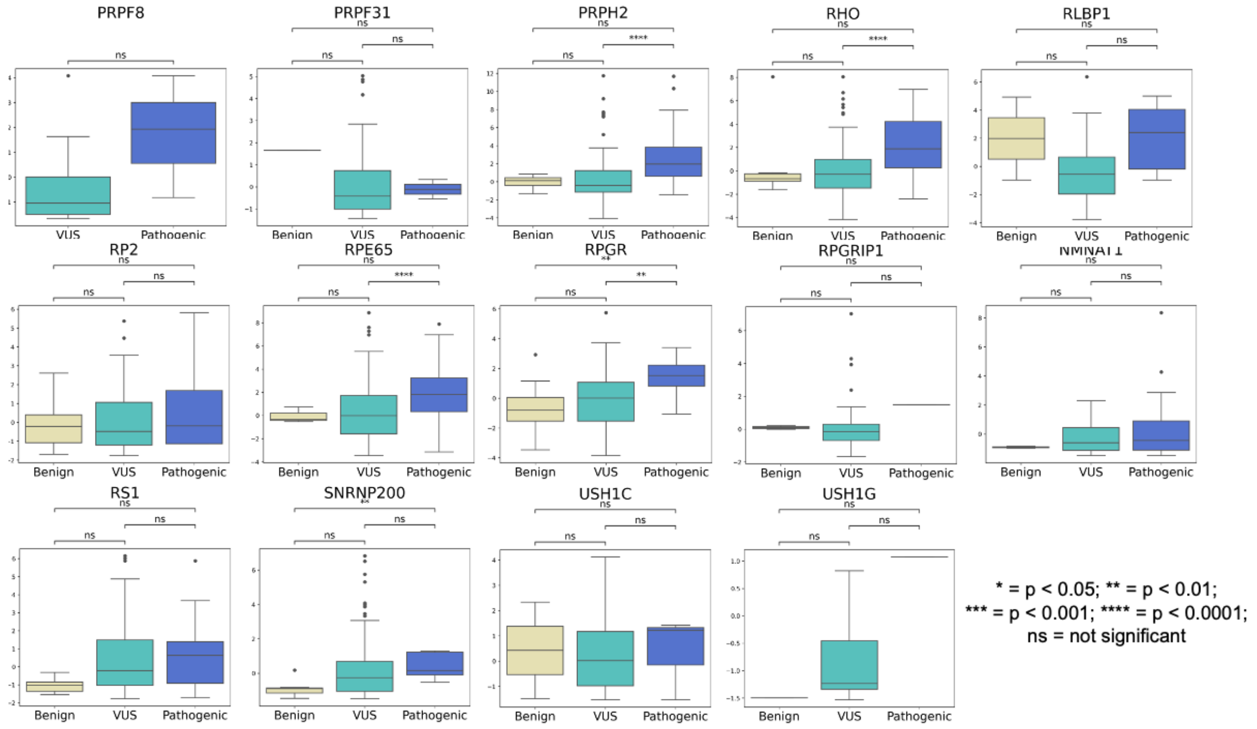

Supplementary Figure 2. Structure-based network analysis highlights pathogenic variants in individual inherited retinal disease proteins. Select individual comparisons between network scores for variants with available clinical phenotype data for inherited retinal disease proteins.

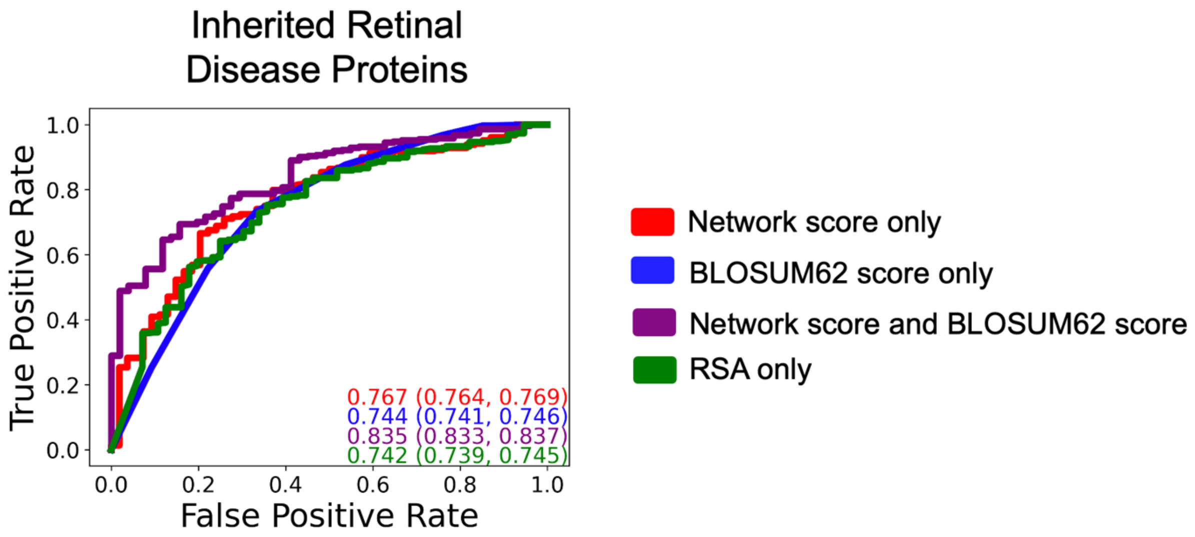

Supplementary Figure 3. Logistic regression-based modelling using SBNA is superior to modelling using RSA alone. Application of univariate and multivariable logistic regression to the inherited retinal disease protein datasets. All regressions were performed using a 70%/30% train/test data split with 500 iterations, and a representative ROC curve with AUC closest to the mean is shown for each regression model. AUC values are displayed as the mean followed by a 95% confidence interval.

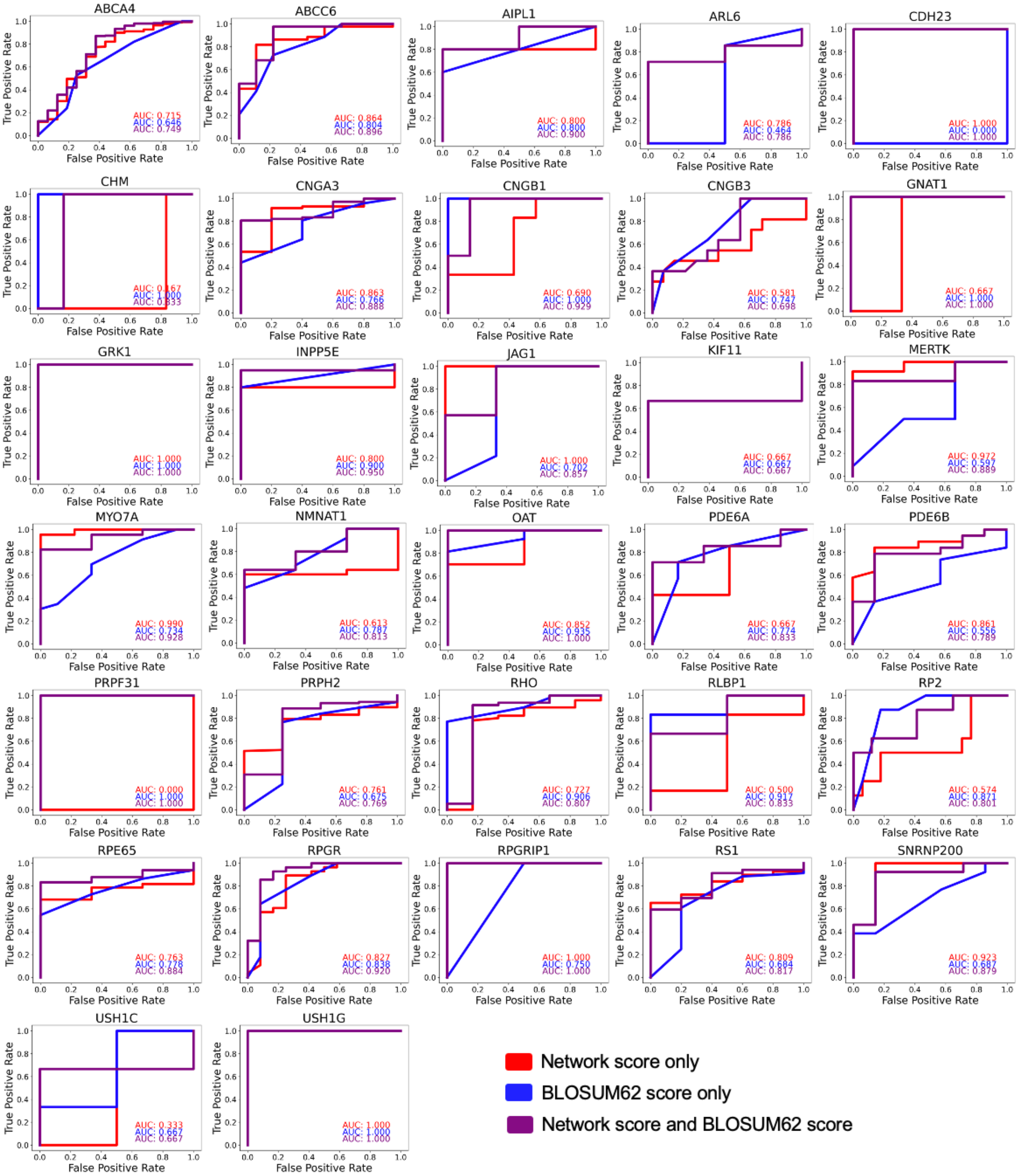

Supplementary Figure 4. Logistic regression-based modelling using SBNA and BLOSUM62 is superior to univariate models for some individual proteins. Application of univariate and multivariable logistic regression models to the 32 inherited retinal disease

proteins for which there was sufficient data to facilitate individual analysis. All regressions were trained on all proteins except the protein of interest and then tested on that protein. ROC curves and AUC values are shown.

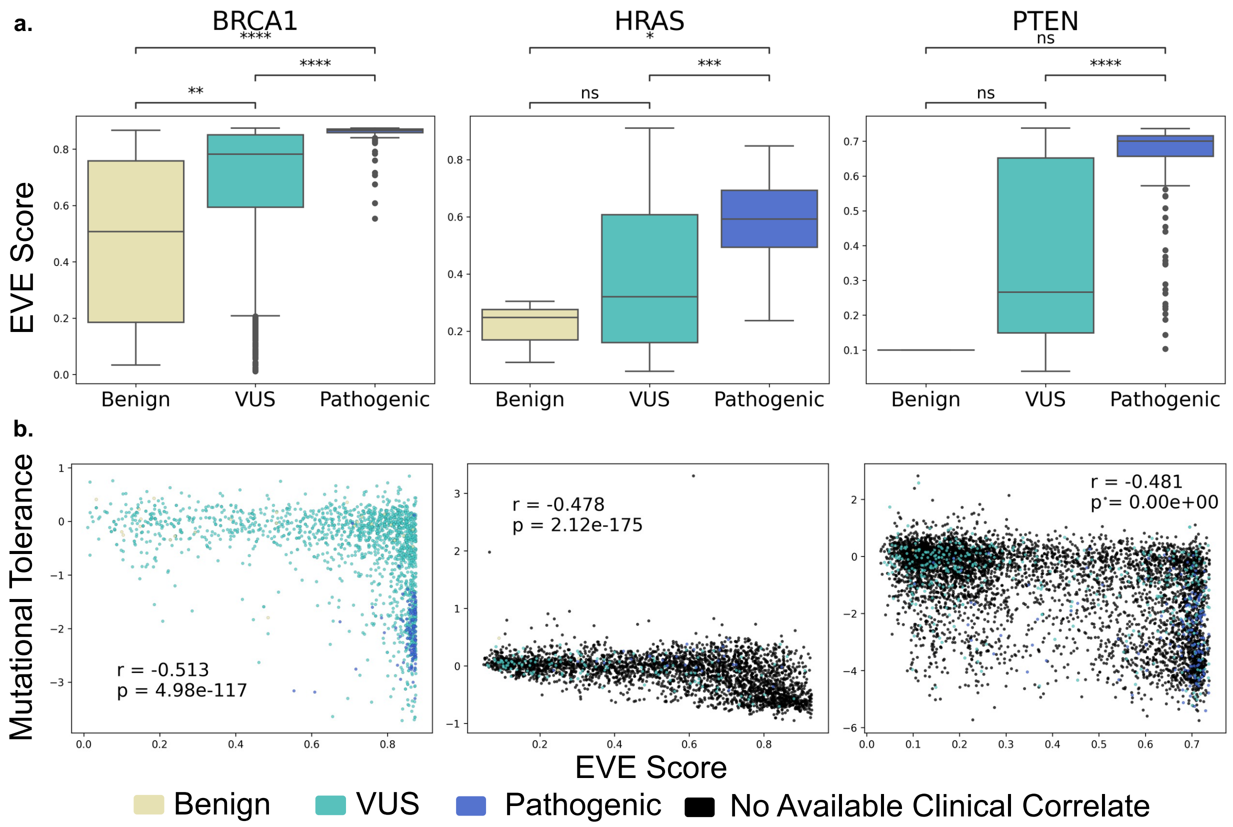

Supplementary Figure 5. EVE score correlates with mutational intolerance. (A) Pooled comparison between EVE scores for variants with available clinical phenotype data. **(B)** Comparison between functional data from saturation mutagenesis experiments and EVE scores, with Spearman correlation coefficients and p-values displayed for each plot. Points are colored based on available clinical phenotype data. EVE score data was not available for *ERK2*, so it was excluded from this analysis.

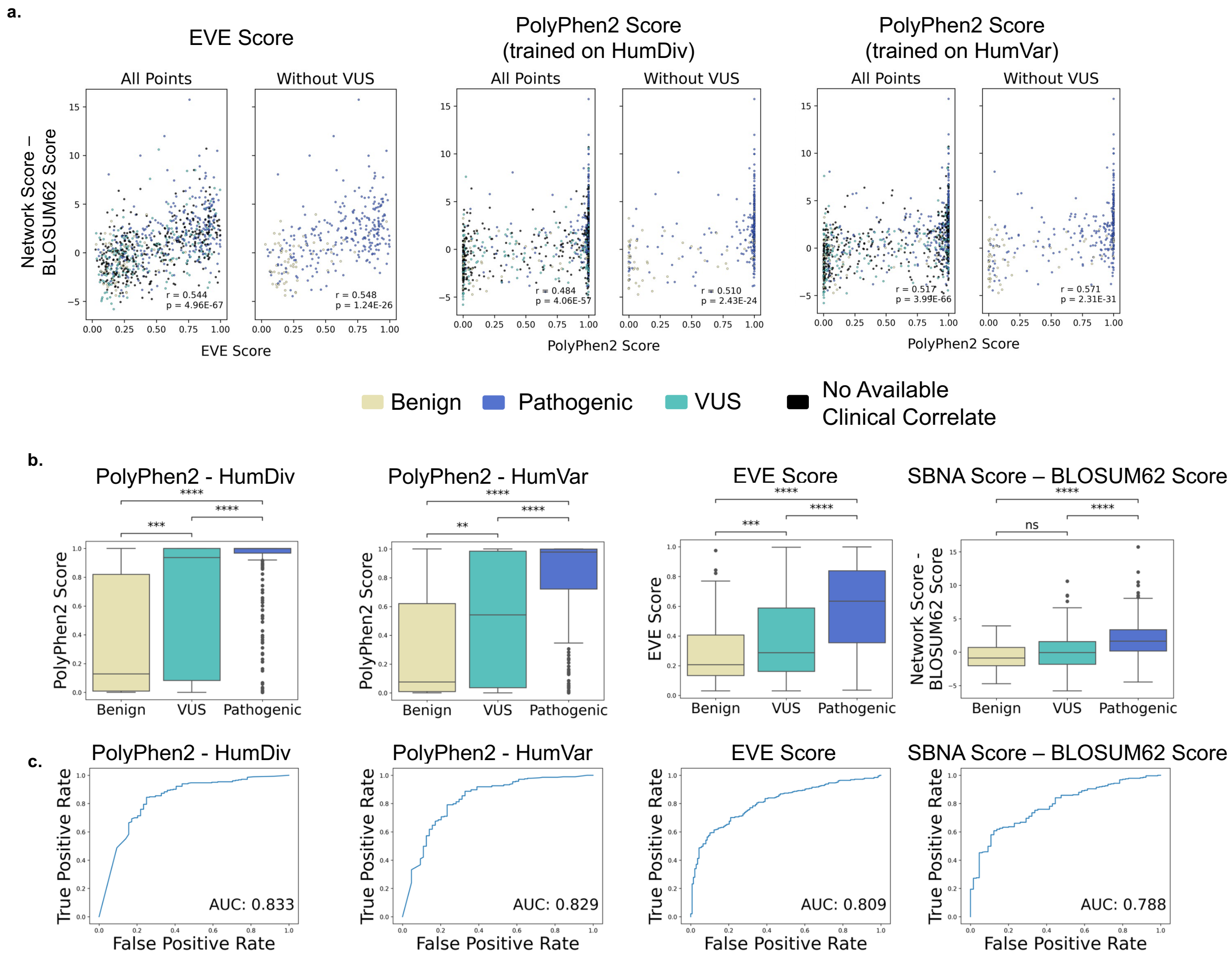

Supplementary Figure 6. Metrics incorporating SBNA scores show similar trends to estimates from PolyPhen2 and EVE within the dataset of all rare variants from patients with IRD at MEE. PolyPhen2[1], EVE[2], and SBNA scores were generated for the full set of all rare variants across patients with IRD at MEE. For each model, the subset of variants for which scores could be calcuated were considered for downstream analysis. **(A)** Comparison between pathogenicity probability estimates generated by PolyPhen2 trained on either the HumDiv or HumVar training data, EVE, and BLOSUM62 scores subtracted from raw SBNA scores with Spearman correlation coefficients displayed for each plot. **(B)** Comparison between pathogenicity probability estimates grouped by benign, VUS, and pathogenic variants as determined by ClinVar and gnomAD. (ns = not significant; * = p<0.05; ** = p<0.01; *** =

p<0.001; **** = p<0.0001) **(C)** ROC curves and AUC values for application of each model. Benign and pathogenic variants were determined based on ClinVar and gnomAD.

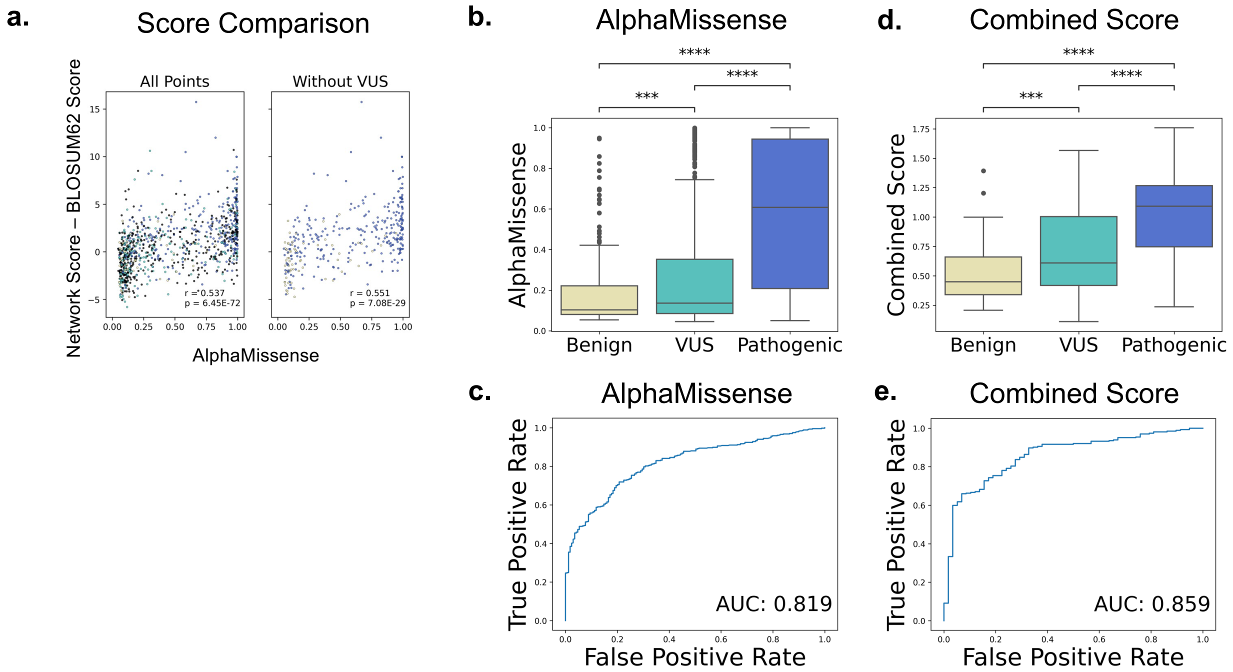

Supplementary Figure 7. Pathogenicity estimates incorporating orthogonal metrics show superior performance. AlphaMissense[3] scores and scores combining the difference between SBNA scores and BLOSUM62 scores as well as EVE[2] scores were generated for the full set of all rare variants across patients with IRD at MEE. The subset of variants for which each score could be calcuated were considered for downstream analysis. **(A)** Comparison between pathogenicity estimates generated by AlphaMissense and BLOSUM62 scores subtracted from raw SBNA scores with Spearman correlation coefficients displayed for each plot. **(B, D)** Comparison between model estimates grouped by benign, VUS, and pathogenic variants as determined by ClinVar and gnomAD. (ns = not significant; * = p<0.05; ** = p<0.01; *** = p<0.001; **** = p<0.0001) **(C, E)** ROC curves and AUC values for application of models. Benign and pathogenic variants were determined based on ClinVar and gnomAD.

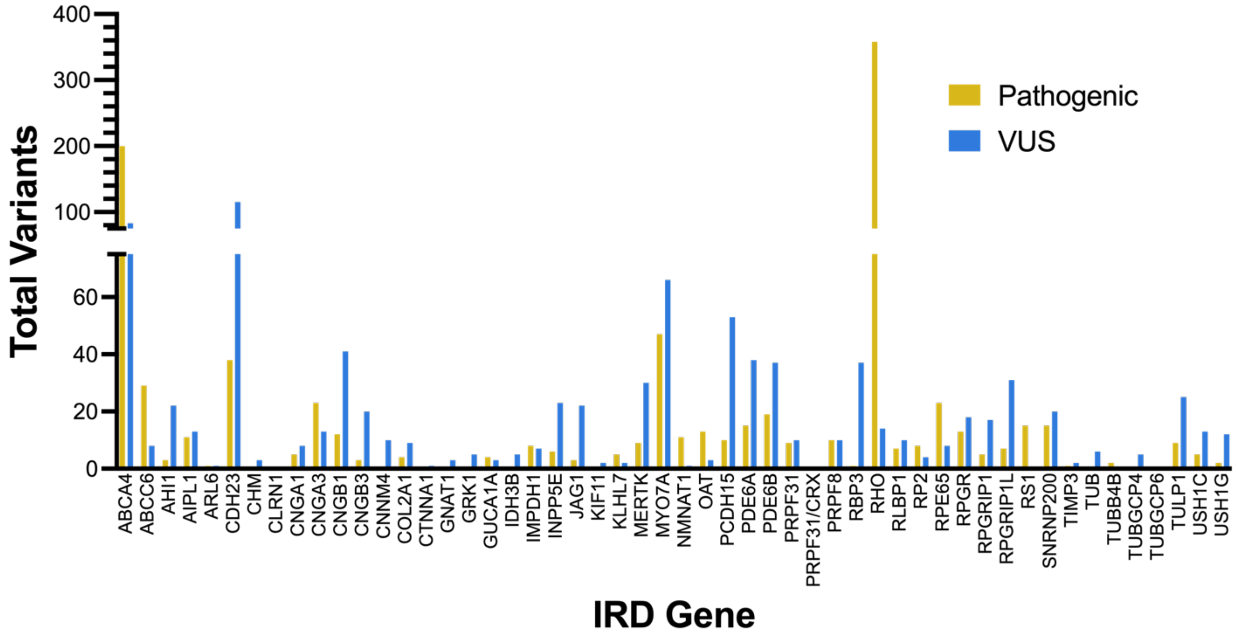

Supplementary Figure 8. Pathogenic variants and VUS from MEE patients span 52 IRD genes. Distribution of total pathogenic variants and VUS (as categorized by ClinVar) from MEE patients across the 52 IRD genes considered in this analysis.

a. b.

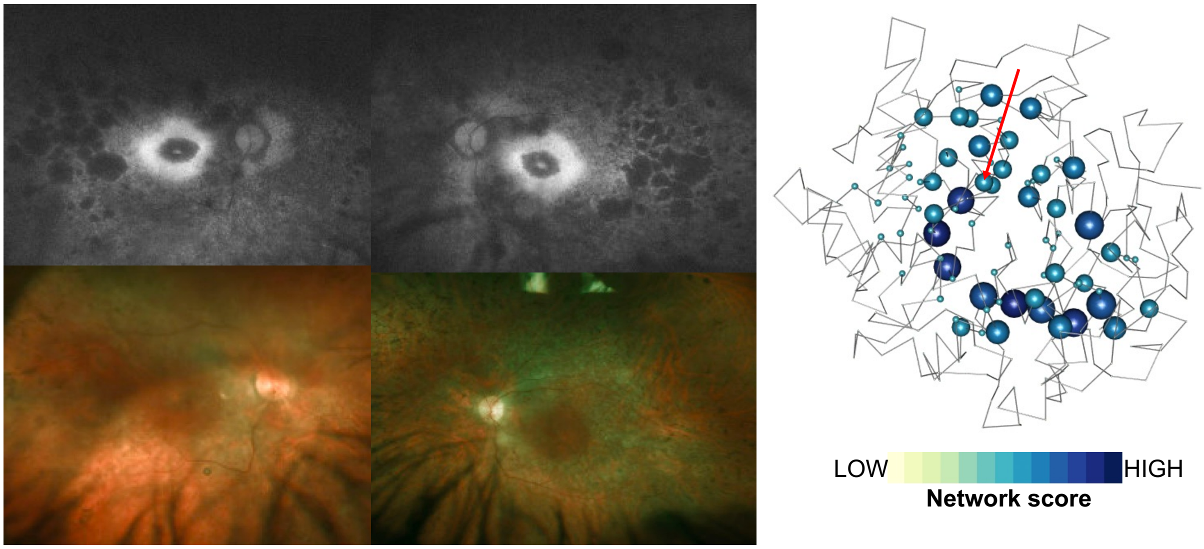

Supplementary Figure 9. SBNA helps identify pathogenic variants in a patient with RPGR-related inherited retinal disease. Representation of network scores for a sample structure with putative solving genetic variants. Sphere radius corresponds to network score magnitude at a particular position. A patient with clinical evidence of RPGR-related disease **(A)** but with no complete genetic explanation was fully solved using SBNA which highlighted a hemizygous variant (Cys302Tyr) that score highly in the RPGR protein structure **(B)**.

Protein	PDB
AHI1	4ESR
ARL6	2H57
CNGA1	7LFT
CNNM4	6G52
CNNM4	6RS2
COL2A1	5NIR
GRK1	3C4Z
GUCA1A	2R2I
IMPDH1	7RER
INPP5E	2XSW
JAG1	4CC0
KIF11	1Q0B
KLHL7	3II7
MERTK	7AB0
NMNAT1	1KKU
OAT	1OAT
OFD1	6E0T
PRPF8	3ENB
RLBP1	3HY5
RP2	2BX6
RS1	3JD6
SNRNP200	4KIT
TUB	1S31
TULP1	3C5N
PTEN	1D5R
HRAS	4NIF
ABCA4	7LKZ
ABCC6	6BZS
ABCC6	6BZR
AIPL1	6PX0
CDH23	5TFM
CDH23	5WJ8
CDH23	5VVM
RPGR	4QAM
RPGRIP1	4QAM
CTNNA1	4IGG
CHM	1VG9
CHM	1VG0

CNGA3	7RHS
CNGB1	7RH9
CNGB3	7RHS
DFNB31	6KZ1
DFNB31	6FDD
DFNB31	6FDE
ERCC6	7OO3
GNAT1	1TND
GNAT1	1TAD
GNAT1	1TAG
IDH3B	6KDF
MYO7A	5MV9
MYO7A	3PVL
PCDH15	5ULY
PCDH15	6E8F
PCDH15	5T4M
PCDH15	4XHZ
PDE6A	6MZB
PDE6B	6MZB
PRPF3	6QW6
PRPF31	2OZB
RBP3	1J7X
RBP3	4LUR
TIMP3	3CKI
USH1C	3K1R
USH1G	3K1R
BRCA1	1JM7
BRCA1	1T29
ERK2	4FMQ
ERK2	4NIF
RPE65	3FSN
RPE65	3KVC
RPE65	4F2Z
RHO	1GZM
RHO	3CAP
PRPH2	7ZW1

Supplementary Table 1. Protein Data Bank accession numbers for well-studied human proteins and IRD genes. Protein Data Bank[4] accession numbers listed here were used to access structural data for well-studied human proteins and IRD proteins.

Supplementary References

1 Adzhubei, I., Jordan, D. M. & Sunyaev, S. R. Predicting functional effect of human missense mutations using PolyPhen-2. *Curr Protoc Hum Genet* **Chapter 7**, Unit7 20, doi:10.1002/0471142905.hg0720s76 (2013).

2 Frazer, J. *et al.* Disease variant prediction with deep generative models of evolutionary data. *Nature* **599**, 91-95, doi:10.1038/s41586-021-04043-8 (2021).

3 Cheng, J. *et al.* Accurate proteome-wide missense variant effect prediction with AlphaMissense. *Science* **381**, eadg7492, doi:10.1126/science.adg7492 (2023).

4 Berman, H. M. *et al.* The Protein Data Bank. *Nucleic Acids Res* **28**, 235-242, doi:10.1093/nar/28.1.235 (2000).
