# Supplementary material for: Structure-based network analysis predicts pathogenic variants in human proteins associated with inherited retinal disease
Source: NPJ Genom Med. 2024 May 27;9:31. doi: 10.1038/s41525-024-00416-w (PMC11130145; doi:10.1038/s41525-024-00416-w)
Supplement: Supplementary file 2 — Reporting summary [file 41525_2024_416_MOESM2_ESM.pdf]

Corresponding author(s): Elizabeth J. Rossin, MD, PhD

Last updated by author(s): Mar 7, 2024

Reporting Summary

Nature Portfolio wishes to improve the reproducibility of the work that we publish. This form provides structure for consistency and transparency in reporting. For further information on Nature Portfolio policies, see our Editorial Policies and the Editorial Policy Checklist.

Statistics

For all statistical analyses, confirm that the following items are present in the figure legend, table legend, main text, or Methods section.

n/a	Confirmed	
☐	☒	The exact sample size (n) for each experimental group/condition, given as a discrete number and unit of measurement
☐	☒	A statement on whether measurements were taken from distinct samples or whether the same sample was measured repeatedly
☐	☒	The statistical test(s) used AND whether they are one- or two-sided<br>*Only common tests should be described solely by name; describe more complex techniques in the Methods section.*
☐	☒	A description of all covariates tested
☐	☒	A description of any assumptions or corrections, such as tests of normality and adjustment for multiple comparisons
☐	☒	A full description of the statistical parameters including central tendency (e.g. means) or other basic estimates (e.g. regression coefficient) AND variation (e.g. standard deviation) or associated estimates of uncertainty (e.g. confidence intervals)
☐	☒	For null hypothesis testing, the test statistic (e.g. F, t, r) with confidence intervals, effect sizes, degrees of freedom and P value noted<br>*Give P values as exact values whenever suitable.*
☒	☐	For Bayesian analysis, information on the choice of priors and Markov chain Monte Carlo settings
☒	☐	For hierarchical and complex designs, identification of the appropriate level for tests and full reporting of outcomes
☐	☒	Estimates of effect sizes (e.g. Cohen's d, Pearson's r), indicating how they were calculated

Our web collection on statistics for biologists contains articles on many of the points above.

Software and code

Policy information about availability of computer code

Data collection	No software was used for data collection.
Data analysis	Data analysis was performed using python and R. Code for structure-based network analysis is publicly available via Zenodo (https://doi.org/10.5281/zenodo.2597484).

For manuscripts utilizing custom algorithms or software that are central to the research but not yet described in published literature, software must be made available to editors and reviewers. We strongly encourage code deposition in a community repository (e.g. GitHub). See the Nature Portfolio guidelines for submitting code & software for further information.

Data

Policy information about availability of data

All manuscripts must include a data availability statement. This statement should provide the following information, where applicable:

- Accession codes, unique identifiers, or web links for publicly available datasets
- A description of any restrictions on data availability
- For clinical datasets or third party data, please ensure that the statement adheres to our policy

ClinVar, gnomAD, and Protein Data Bank data are publicly available and can be accessed using the gene names and unique identifiers in Table S1. Patient data from Massachusetts Eye and Ear cannot be shared publicly due to concerns regarding possible identifiability of patients with relatively rare genetic conditions.

Research involving human participants, their data, or biological material

Policy information about studies with human participants or human data. See also policy information about sex, gender (identity/presentation), and sexual orientation and race, ethnicity and racism.

Reporting on sex and gender	Reporting on sex was considered from the perspective of genotypic analysis (e.g., considering whether a patient had only one X-chromosome and might therefore be susceptible to X-linked recessive conditions).
Reporting on race, ethnicity, or other socially relevant groupings	This data was not collected for patients included in the Massachusetts Eye and Ear dataset.
Population characteristics	Genotypic information
Recruitment	Patient data was gathered from among those presenting to the Inherited Retinal Disorders Service at Massachusetts Eye and Ear between the 1980s and 2020s.
Ethics oversight	Appropriate consent was obtained from all included patients, and approval was granted by the Mass General Brigham/ Massachusetts Eye and Ear Institutional Review Board Protocol Number 2019P001098. This study was approved by the local institutional review board and adhered to the Declaration of Helsinki. Informed consent was obtained from all individuals on whom clinical data and genetic testing were performed.

Note that full information on the approval of the study protocol must also be provided in the manuscript.

Field-specific reporting

Please select the one below that is the best fit for your research. If you are not sure, read the appropriate sections before making your selection.

☒ Life sciences ☐ Behavioural & social sciences ☐ Ecological, evolutionary & environmental sciences

For a reference copy of the document with all sections, see nature.com/documents/nr-reporting-summary-flat.pdf

Life sciences study design

All studies must disclose on these points even when the disclosure is negative.

Sample size	No sample size calculation was performed as this was not a clinical trial
Data exclusions	Patients without available genotypic information were not included in this study.
Replication	N/A, no intervention was tested in this study.
Randomization	Randomization was not relevant to the prospectively collected cohort of patients in this study.
Blinding	N/A, no intervention was tested in this study.

Reporting for specific materials, systems and methods

We require information from authors about some types of materials, experimental systems and methods used in many studies. Here, indicate whether each material, system or method listed is relevant to your study. If you are not sure if a list item applies to your research, read the appropriate section before selecting a response.

Materials & experimental systems

n/a	Involved in the study	
☒	☐	Antibodies
☒	☐	Eukaryotic cell lines
☒	☐	Palaeontology and archaeology
☒	☐	Animals and other organisms
☐	☒	Clinical data
☒	☐	Dual use research of concern
☒	☐	Plants

Methods

n/a	Involved in the study	
☒	☐	ChIP-seq
☒	☐	Flow cytometry
☒	☐	MRI-based neuroimaging

Clinical data

Policy information about clinical studies

All manuscripts should comply with the ICMJE guidelines for publication of clinical research and a completed CONSORT checklist must be included with all submissions.

Clinical trial registration	N/A
Study protocol	N/A
Data collection	Patient data was gathered from among those presenting to the Inherited Retinal Disorders Service at Massachusetts Eye and Ear between the 1980s and 2020s.
Outcomes	Patient outcomes were not assessed in this study.

Plants

Seed stocks	N/A
Novel plant genotypes	N/A
Authentication	N/A
